# irGSEA: the integration of single-cell rank-based gene set enrichment analysis

**DOI:** 10.1093/bib/bbae243

**Published:** 2024-05-27

**Authors:** Chuiqin Fan, Fuyi Chen, Yuanguo Chen, Liangping Huang, Manna Wang, Yulin Liu, Yu Wang, Huijie Guo, Nanpeng Zheng, Yanbing Liu, Hongwu Wang, Lian Ma

**Affiliations:** Department of Hematology and Oncology, Shenzhen Children's Hospital of China Medical University, Shenzhen 518038, China; Department of Obstetrics and Gynecology; Department of Pediatrics; Guangdong Provincial Key Laboratory of Major Obstetric Diseases; Guangdong Provincial Clinical Research Center for Obstetrics and Gynecology; Guangdong-Hong Kong-Macao Greater Bay Area Higher Education Joint Laboratory of Maternal-Fetal Medicine, The Third Affi1iated Hospital of Guangzhou Medical University, Guangzhou 510150, China; Department of Hematology and Oncology, Shenzhen Children's Hospital of China Medical University, Shenzhen 518038, China; Department of Hematology and Oncology, Shenzhen Children's Hospital of China Medical University, Shenzhen 518038, China; Department of Obstetrics and Gynecology; Department of Pediatrics; Guangdong Provincial Key Laboratory of Major Obstetric Diseases; Guangdong Provincial Clinical Research Center for Obstetrics and Gynecology; Guangdong-Hong Kong-Macao Greater Bay Area Higher Education Joint Laboratory of Maternal-Fetal Medicine, The Third Affi1iated Hospital of Guangzhou Medical University, Guangzhou 510150, China; Department of Pediatrics, The Second Affiliated Hospital of Shantou University Medical College, Shantou 515041, China; Department of Hematology and Oncology, Shenzhen Children's Hospital of China Medical University, Shenzhen 518038, China; Department of Hematology and Oncology, Shenzhen Children's Hospital of China Medical University, Shenzhen 518038, China; Department of Pediatrics, The Second Affiliated Hospital of Shantou University Medical College, Shantou 515041, China; Department of Hematology and Oncology, Shenzhen Children's Hospital of China Medical University, Shenzhen 518038, China; Department of Pediatrics, The Second Affiliated Hospital of Shantou University Medical College, Shantou 515041, China; Department of Obstetrics and Gynecology; Department of Pediatrics; Guangdong Provincial Key Laboratory of Major Obstetric Diseases; Guangdong Provincial Clinical Research Center for Obstetrics and Gynecology; Guangdong-Hong Kong-Macao Greater Bay Area Higher Education Joint Laboratory of Maternal-Fetal Medicine, The Third Affi1iated Hospital of Guangzhou Medical University, Guangzhou 510150, China; Department of Hematology and Oncology, Shenzhen Children's Hospital of China Medical University, Shenzhen 518038, China

**Keywords:** single-cell RNA sequencing, rank-based gene set enrichment analysis, robust rank aggregation algorithm

## Abstract

irGSEA is an R package designed to assess the outcomes of various gene set scoring methods when applied to single-cell RNA sequencing data. This package incorporates six distinct scoring methods that rely on the expression ranks of genes, emphasizing relative expression levels over absolute values. The implemented methods include AUCell, UCell, singscore, ssGSEA, JASMINE and Viper. Previous studies have demonstrated the robustness of these methods to variations in dataset size and composition, generating enrichment scores based solely on the relative gene expression of individual cells. By employing the robust rank aggregation algorithm, irGSEA amalgamates results from all six methods to ascertain the statistical significance of target gene sets across diverse scoring methods. The package prioritizes user-friendliness, allowing direct input of expression matrices or seamless interaction with Seurat objects. Furthermore, it facilitates a comprehensive visualization of results. The irGSEA package and its accompanying documentation are accessible on GitHub (https://github.com/chuiqin/irGSEA).

## Graphical Abstract

**Graphical Abstract:**
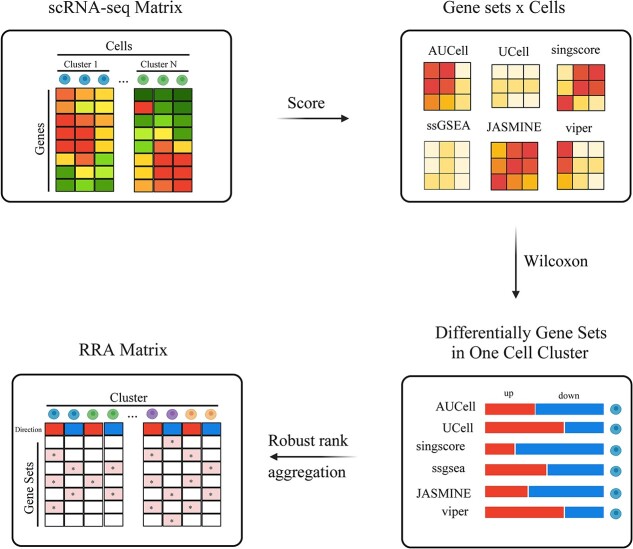

The workflow of irGSEA. Individual cells were independently scored using AUCell, UCell, singscore, ssGSEA, JASMINE and Viper. Differential gene sets were calculated for each cell cluster using distinct enrichment score matrices, with significance determined at an adjusted *P*-value of ≤ 0.05 (Wilcoxon test). The ‘Up’ or ‘Down’ designation indicates whether the enrichment degree of the differential gene set in the cell cluster is higher or lower compared to other clusters. The robust rank aggregation algorithm was employed to filter statistically significant gene sets that exhibited similar levels of enrichment across all six methods.

## Introduction

Signature gene sets are vital constructs derived from the gene expression analysis across diverse cell types or biological states, facilitated by technologies such as microarrays, bulk transcriptome sequencing and proteomics [1]. These sets play a pivotal role in discerning cell types within unknown clusters and elucidating their biological functions through single-cell RNA sequencing. Gene set enrichment analysis emerges as an effective method for linking signature gene sets to these unknown clusters. The degree of enrichment in a gene set signifies the strength of the connection between the biological functions represented by the set and a given unknown cluster.

Presently, three primary methods are employed for gene set enrichment analysis: over-representation analysis (ORA), functional class scoring (FCS) and pathway topology analysis (PTA) [2]. ORA is constrained by its reliance on threshold filtering of differentially expressed genes and low folding, potentially leading to the exclusion of significant genes. In contrast, PTA faces limitations due to the scarcity of pathway topology information in most public databases or studies. Consequently, FCS stands out as the preferred method for gene set enrichment analysis, as it can accommodate low-fold change genes and incomplete pathway topology information [2].

We conducted a review of 17 common FCS methods, encompassing gene set enrichment analysis (GSEA) [3], gene set variation analysis (GSVA) [4], pathway-level analysis of gene expression (PLAGE) [5], zscore [6], addModuleScore [7], single-cell signature explorer (SCSE) [8], Vision [9], variance-adjusted Mahalanobis (VAM) [10], gficf [11], pagoda2 [12], AUCell [13], UCell [14], singscore [15], single sample gene set enrichment analysis (ssGSEA) [4], jointly assessing signature mean and inferring enrichment (JASMINE) [16], virtual inference of protein activity by enriched regulon analysis (Viper) [17] and Sargent [18]. GSEA detects gene set enrichment at the top or bottom of a ranked gene list, calculated based on all cell clusters using the ranked gene signal-to-noise ratio or ranked gene fold-change. GSVA estimates the kernel of the cumulative density function for each gene between all cells. PLAGE standardizes the gene expression matrix across cells and extracts singular-value decompositions as enrichment scores. The *z*-score aggregates the expression of all genes within a gene set, scaling gene expression using standard deviations over cells. AddModuleScore partitions the cell expression matrix based on the mean of all genes in a gene set and filters out control genes outside the set to determine background values. SCSE quantifies a signature gene set using the normalized total expression of its genes. Vision averages gene expression within each set, correcting enrichment scores using means and standard deviations. VAM generates scores from single-cell RNA sequencing data based on variations in classic Mahalanobis multivariate distance measures. Gficf capitalizes on informative biological signals across latent factors of gene expression values obtained from nonnegative matrix factorization. Pagoda2 fits an error model for each cell, quantifying enrichment scores using the first weighted principal component. AUCell uses the area under the curve (AUC) to assess whether a gene set is enriched within the top 5% of expressed genes for each cell based on gene expression rank. The AUC represents the enrichment score of the gene set. UCell computes a ranked list of genes for each cell, exclusively considering the top 1500 genes to address uninformative tails, and computes the Mann–Whitney U statistic for gene set enrichment. SingScore assesses distance from the center of individual cells based on gene expression rank, normalizing mean ranks and aggregating them to represent the enrichment score. ssGSEA calculates differential scores in the empirical cumulative distribution between internal and external gene sets based on cell gene expression rank, normalizing to global expression profiles. JASMINE approximates mean values based on gene ranks within expressed genes and normalizes both the mean and gene set enrichment values to yield a final score. Viper estimates the enrichment score of a gene set by performing a three-tailed calculation based on the rank of gene expression across cells. Sargent transforms gene expression data into a gene-set-by-cell assignment score matrix, calculating a Gini index among assignment scores per cell.

Numerous FCS methods, including GSEA, GSVA, PLAGE, addModuleScore, SCSE, Vision, VAM, gficf, pagoda2 and Sargent, consider the composition of the dataset. Changes in dataset composition influence the enrichment fractions of identical cells. When integrating new single-cell datasets into existing data, it becomes necessary to recalculate gene set enrichment scores for every cell using these FCS methods. This process can be both tedious and resource-intensive. In contrast, gene set scoring based on individual cell expression ranks, such as AUCell, UCell, singscore, ssGSEA, JASMINE and Viper, requires computation of the enrichment score solely for the added single-cell dataset. This is because the enrichment scores generated by these methods depend solely on the relative gene expression at the individual cell level and are independent of the dataset composition. Consequently, these methods significantly save time.

Single enrichment analysis methods offer limited information, prompting the exploration of combining multiple methods. The amalgamation of methods demonstrated superior performance over individual methods in gene set scoring [19]. However, a simple intersection of differential results among gene set scoring methods yielded conservative outcomes and overlooked vital information, such as the relative enrichment degree among gene sets. To address this limitation, our approach involves comprehensively judging a gene set that is enriched across multiple scoring methods using the robust rank aggregation algorithm (RRA). We aggregated results from diverse methods, including AUCell, UCell, singscore, ssGSEA, JASMINE and Viper. For user-friendly implementation, we developed the R package irGSEA (https://github.com/chuiqin/irGSEA/), which integrates the described workflow and presents evaluation results through various visualizations.

Our study employed a comprehensive enrichment analysis that integrated multiple methods to overcome the limitations associated with single-method analyses. Previous research has established that the combination of multiple methods enhances the accuracy of gene set scoring [19]. However, a simple intersection of differential results from all gene set scoring methods yielded conservative outcomes and overlooked crucial information, such as the relative enrichment degree among gene sets. To address this limitation, we chose to aggregate results from various methods, including AUCell, UCell, singscore, ssGSEA, JASMINE and Viper, to comprehensively assess gene sets exhibiting high levels of enrichment across multiple scoring methods. We utilized the robust RRA to focus on gene sets that demonstrated consistent levels of enrichment across multiple scoring methods. To facilitate this process, we developed the user-friendly R package irGSEA (https://github.com/chuiqin/irGSEA/), enabling the implementation of the workflow and visualization of results. The primary objective of the irGSEA package is to aid users in evaluating potentially significant biological processes within cell clusters. These biological processes were identified in many FCS methods, including AUCell, UCell, singscore, ssGSEA, JASMINE and Viper.

## Method

### Rank-based GSEA

We employed six rank-based gene set scoring methods: AUCell, UCell, singscore, ssGSEA, JASMINE and Viper. To facilitate single-cell GSEA, we modified ssGSEA by excluding the final normalization step. These methods were implemented using R packages, specifically AUCell (version 1.14.0), UCell (version 1.1.0), singscore (version 1.12.0), GSVA (version 1.40.1) and Viper (version 1.32.0).

### Construction of input gene sets

This process enables users to access predefined gene sets from MSigDB using the built-in R package MSigDB (version 7.4.1) [20]. MSigDB incorporates thousands of annotated gene sets, covering various categories such as hallmark gene sets—coherently expressed signatures derived by aggregating multiple MSigDB gene sets to represent well-defined biological states or processes. It also includes KEGG, Wiki, Reactome, and Gene Ontology gene sets (encompassing GO Biological Process ontology, GO Cellular Component ontology and GO Molecular Function ontology). By utilizing the predefined gene sets available in the MSigDB database, users could quickly identify the potential biological functions of the cell cluster. Users could enter multiple species (encompassing *Homo sapiens*, *Mus musculus*, *Rattus norvegicus*, etc.) or gene formats (encompassing Gene Symbol, Entrez Gene ID and Ensembl Gene ID). Additionally, users could have the option to create their own gene sets for enrichment analysis.

### Cleaning of input objects

Single-cell expression matrices or Seurat objects can be directly inputted. Gene set enrichment score matrices were stored in the Seurat object using the R package Seurat (version 4.0.3) [7]. Users can filter genes with zero expression or apply customized filtering criteria for single-cell expression matrices.

### Comprehensive assessment

Individual cells were scored separately using multiple gene set scoring methods. The Wilcoxon test was employed to assess the differential gene sets of the cell clusters across various scoring matrices. The criterion for filtering statistically significant differential gene sets was that the adjusted *P*-value should be less than or equal to 0.05. The *P*-value adjustment was performed using Bonferroni correction, executed through the FindMarker function of the Seurat R package.

Subsequently, RRA was performed. The first step in the RRA is to calculate the standardized rank:


$$ {r}_{ij}={R}_{ij}/ Ma{x}_{j=1}^k\left({R}_{ij}\right) $$

$i$ is the significant target gene set, $j$ is the scoring method and $k$ is the number of scoring methods used. ${r}_{ij}$ is the standardized rank of the significant gene set $i$ in the scoring method $j$. ${R}_{ij}$ is the original rank of the significant gene set $i$ in the scoring method $j$. ${\mathit{\operatorname{Max}}}_{j=1}^k\left({R}_{ij}\right)$ is the largest original rank among $k$ types of scoring methods.

We sorted ${r}_{ij}$ from small to large to generate ${r}_{(i)=\left({r}_{(1)},\dots, {r}_{(k)}\right)}$. Next, we randomly sampled from the uniform distribution to generate the same number of null models and sorted them from small to large to generate ${r}_{\left(i, null\right)=\left({r}_{\left(1, null\right)},\dots, {r}_{\left(k, null\right)}\right)}$. We also define ${\beta}_{x,k}\left({r}_{(x)}\right)$ as the probability of ${r}_{\left(x, null\right)\le }\ {r}_{(x)}$:


$$ {\beta}_{x,k}\left({r}_{(x)}\right):= {\sum}_{\ell =x}^{\mathrm{k}}\left(\begin{array}{c}\mathrm{k}\\{}\ell \end{array}\right)\ {\left({\mathrm{r}}_{\left(\mathrm{x}\right)}\right)}^{\ell }{\left(1-\left({r}_{(x)}\right)\right)}^{k-\ell }, $$

and generate the same number of probabilities ${\beta}_{\left(\mathrm{i}\right)}=({\beta}_{1,k}\left({r}_{(1)}\right),\dots, {\beta}_{k,k}\left({r}_{(k)}\right))$. ${\beta}_{\left(\mathrm{i}\right)}$is a vector. We define the score of the robust RRA as the minimum value of ${\beta}_{\left(\mathrm{i}\right)}$, and the score was corrected by Bonferroni correction against bias coming from multiple hypothesis testing. A *P*-value ≤0.05 was considered statistically significant. This flow was implemented using the R package RobustRankAggreg (version 1.1.0) [21].

### Visualization

Several visualization functions were created to present comprehensive evaluation outcomes and target gene distributions from specific enrichment analyses. The Complex Heatmap package (version 2.8.0) [22] was used to construct heatmaps, upset plots and density heatmaps. Bubble and stack bar plots were generated using the ggplot2 (version 3.3.5) [23], ggtree (version 3.0.2) [24] and aplot packages (version 3.0.2) [25]. The density scatter plot was constructed using the Nebulosa package (version 1.2.0) [26], whereas the half-violin and ridge plots were constructed using the gghalves package (version 0.1.1) [27] and ggridges package (version 0.5.3) [28], respectively. Eventually, these plots were converted into ggplot2 objects using the ggplotify package (version 0.0.7) [29], which facilitated their combination with other ggplot2 objects.

### Statistical analysis

The Wilcoxon test was used to assess the differential gene sets of the cell clusters across various scoring matrices. The *P*-value adjustment was performed using Bonferroni correction. Statistical significance was set at an adjusted *P*-value ≤ 0.05. Kendall's coefficient of concordance was employed to measure the consistency among the scoring methods.

## Results

### Overview of irGSEA workflow

The irGSEA workflow is depicted in the **Graphical Abstract**, illustrating its application to a single-cell peripheral blood mononuclear cell dataset. We utilized a demo dataset comprising 13 714 genes and 2638 cells processed with 10X GENOMICS. Each cell was assigned to predefined cell types, and the dataset was accessible through the R package SeuratData (version 0.2.1) [30]. This dataset served as a demonstration, showcasing the workflow of the R package irGSEA.

**Figure 1:**
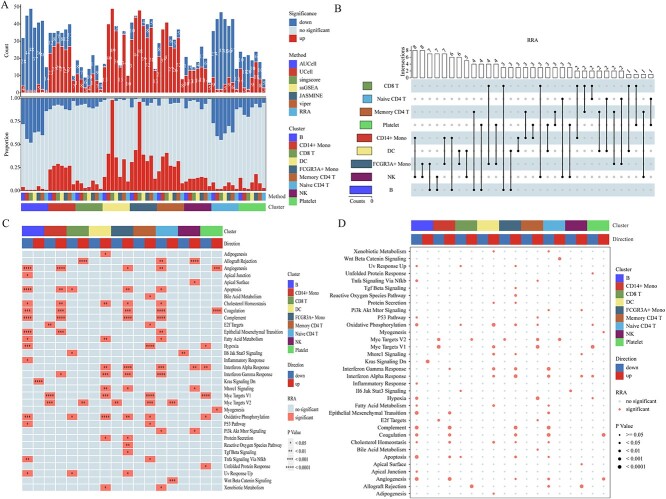
Overall visualization. (**A**) Stack Bar Plot: this comprises two components, illustrating the count of significant gene sets detected by various scoring methods and the total count of significant gene sets identified by RRA. The top bar exhibits the count of significant gene sets for each cluster in differentiating methods. Up or down indicates whether the enrichment degree of the differential gene set in the cell cluster is higher or lower than in other clusters. The middle bar represents the percentage of significant (up and down) and insignificant gene sets for each cell cluster in different scoring methods (**B**) UpSet Plot: Visualizes the overlap of differential gene sets identified by RRA across various pairwise cell clusters. The left bar represents different clusters, and the number of overlapping intersections is depicted by the bar above. (**C**) Heatmap: visualizes the distribution of significant gene sets identified by RRA across distinct cell clusters. The number of asterisks in the grid's upper half indicates the *P*-value. The left clustering tree lays out gene set pattern similarity between clusters. Up or down in the legend named direction indicates whether the enrichment degree of the differential gene set in the cell cluster is higher or lower than in other clusters. (**D**) Bubble Plot: illustrates the distribution of significant gene sets identified by RRA among different cell clusters. The clustering tree on the left represents the similarity of gene set patterns among the clusters. The size of the dots corresponds to the *P*-value. Up or down in the legend named direction indicates whether the enrichment degree of the differential gene set in the cell cluster is higher or lower than in other clusters.

**Figure 2:**
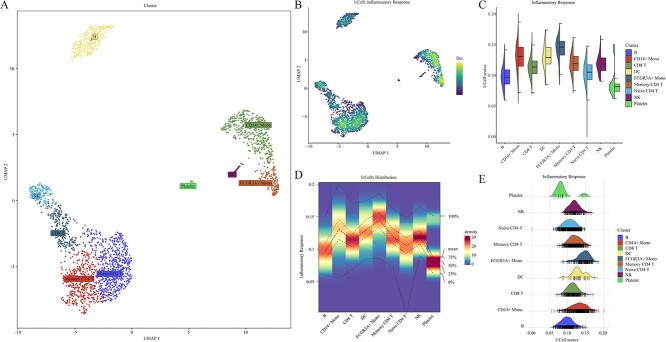
Local visualization. (**A**) UMAP plot illustrating the distribution of distinct cell clusters in a low-dimensional space. (**B**) Density scatterplot transforms the gene set enrichment score into density scores, mapped onto UMAP dimensions. The higher density score indicates increased gene set enrichment in the cell. (**C**) Half violin plot displaying the distribution and expression of the gene set using box plots (right) and violin plots (left), respectively. (**D**) Density heatmap visualizing the distribution and expression of the gene set for different clusters. Quintiles and means of enrichment scores are indicated by dashed lines. (**E**) Ridge plot illustrating the distribution and expression of the gene set across different clusters through kernel density curves (top) and barcode plots (bottom).

**Figure 3:**
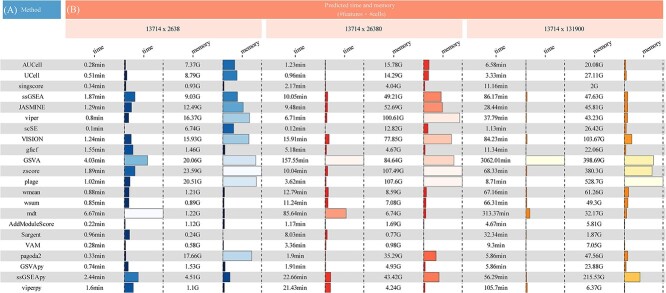
Comparison of different scoring methods. The plot illustrates the time and peak memory consumption associated with 50 Hallmark gene sets across various scoring methods for datasets of varying sizes. GSVApy, ssGSEApy and viperpy denote the Python versions of GSVA, ssGSEA and Viper, respectively. The memory peaks of singscore, ssGSEA, JASMINE and Viper have been optimized. To address memory peak issues for datasets exceeding 50 000 cells, we implemented a strategy of partitioning them into processing units of 5000 cells each for scoring. While this strategy mitigates memory peak issues, it extends the processing time.

To evaluate functionality, the irGSEA score function assigned scores to all cells using 50 hallmark gene sets. Differential gene sets were then calculated using various scoring methods. Subsequently, we employed the irGSEA integrated function to comprehensively assess gene set significance across multiple scoring methods, with the results presented in Fig. 1. The stack bar plot illustrates the count of significant gene sets detected by various scoring methods and the total count of significant gene sets identified by RRA (Fig. 1A). The upset plot visualizes the overlap of differential gene sets identified by RRA across various pairwise cell clusters (Fig. 1B). The heatmap and bubble plots show the distribution of significant gene sets identified by RRA across distinct cell clusters (Fig. 1C–D). Figure 2 illustrates a showcase scenario focusing on the UCell and the ‘Inflammatory Response’ gene set, allowing users to choose this visualization for each incorporated scoring method. The irGSEA score function supports various gene set scoring methods, including GSEA, GSVA, PLAGE, addModuleScore, SCSE, Vision, VAM, gficf and pagoda2. These methods do not rely on the expression rank of individual cells. We augmented the demo dataset 10-fold and 50-fold to evaluate the time and peak memory associated with different gene set scoring methods in irGSEA, aiding users in making informed choices tailored to their private datasets (Fig. 3). We also measure the consistency among the scoring methods, including AUCell, UCell, songscore, ssGSEA, JASMINE and Viper, through Kendall's coefficient of concordance. We calculated the median Kendall's coefficient among these methods in one-, ten-, and fifty-fold demo datasets. The overall Kendall's coefficients among the six methods were low. Three methods designed explicitly for single-cell datasets did not exhibit the highest consistency. ssGSEA and Viper showed the top 1 Kendall's coefficient, while AUCell and Viper showed the leading 2 Kendall's coefficient (Supplementary Table S1). The workflow and code are available on GitHub (2).

## Discussion

Our study outlines a framework for ranking-based single-cell GSEA, wherein only the gene expression rank for individual cells is considered. The scores from AUCell, UCell, singscore, ssGSEA, JASMINE and Viper remained robust against variations in dataset composition. AUCell, UCell and JASMINE represent gene set scoring methods tailored for single-cell transcriptomes, considering the impact of dropout events in individual cells. AUCell and UCell intuitively address dropout effects by focusing on the top few thousand highly expressed genes. JASMINE addresses dropout effects by evaluating the enrichment of signature genes among all expressed genes. Singscore, ssGSEA and Viper are widely used gene set scoring methods designed for bulk transcriptomes. However, bulk sample-based methods are not explicitly crafted to handle substantial dropouts, potentially leading to misguidance in the analysis [16]. Nevertheless, AUCell, UCell and JASMINE exhibited differences in their abilities to identify differential gene sets. An obvious concern is what is the benefit of adding bulk-based scoring methods to the aggregation when the target is single-cell data? We calculated Kendall's coefficient among these six scoring methods in different sizes of single-cell datasets. We measured the consistency among six methods using the median Kendall's coefficient. The overall Kendall's coefficients among the six methods were low, indicating significant differences. Interestingly, three methods designed explicitly for single-cell datasets did not exhibit the highest consistency. ssGSEA and Viper showed the top 1 Kendall's coefficient, while AUCell and Viper showed the leading 2 Kendall's coefficient. The results of the consistency analysis provide empirical evidence that incorporating bulk-based scoring methods can reduce biases introduced by solely including single-cell scoring methods. It also aligns with the purpose of irGSEA, which is to aggregate scoring results from diverse methods to ascertain the statistical significance of target gene sets in most methods to assist researchers in validating their results with greater confidence. Therefore, our framework incorporates the RRA approach to identify significantly enriched gene sets across various scoring methods. The framework integrates several user-friendly visualization tools to display the results and is packaged into the R package irGSEA, which is available on GitHub (https://github.com/chuiqin/irGSEA).

## Conclusion

In this article, we create an R package called irGSEA to amalgamate results from all six methods to ascertain the statistical significance of target gene sets across diverse scoring methods by employing the robust RRA. irGSEA prioritizes user-friendliness, allowing direct input of expression matrices or seamless interaction with Seurat objects. Furthermore, irGSEA integrates several user-friendly visualization tools to display the results. Finally, in future work, we intend to expand the irGSEA into the Python working framework and add more gene set scoring methods. We will refine the scope of irGSEA's inclusion to illustrate that scoring methods can be included under different conditions, such as pseudobulk and imputation.

## Limitations

Although irGSEA works with the R working framework and is user-friendly, it recommends that people who have mastered the basic skills of the R language use it. We have optimized the memory consumption and running time of irGSEA, but calculating 1 million cells is still a challenge. irGSEA also needs to test which scoring methods can be included under different conditions, such as pseudobulk and imputation, to make the best benefit.

**Key Points**

- irGSEA amalgamates results from all six rank-based methods by employing the robust rank aggregation algorithm to ascertain the statistical significance of target gene sets across diverse scoring methods.
- irGSEA supports 19 gene set scoring methods and integrates several user-friendly visualization tools to display the results.
- irGSEA facilitates assessing potentially significant biological processes within cell clusters, emphasizing its utility in exploring cell functionalities rather than excelling in cell type identification.

## Abbreviations

ORA: over-representation analysis

FCS: functional class scoring

PTA: pathway topology analysis

GSEA: gene set enrichment analysis

GSVA: gene set variation analysis

PLAGE: pathway level analysis of gene expression

SCSE: single-cell signature explorer

VAM: variance-adjusted mahalanobis

ssGSEA: single sample gene set enrichment analysis

JASMINE: jointly assessing signature mean and inferring enrichment

Viper: virtual inference of protein activity by enriched regulon analysis

AUC: area under the curve

MSigDB: molecular signatures database

RRA: robust rank aggregation

## Supplementary Material

Supplementary_Table_S1_bbae243

## Data Availability

Additional tables and high-resolution plots are included in the supplementary materials. The workflow or code is available on GitHub (https://chuiqin.github.io/irGSEA/).

## Notes

Biographical Note: As clinicians, we are committed to using bioinformatics to address clinical challenges.
