# Supplementary material for: irGSEA: the integration of single-cell rank-based gene set enrichment analysis
Source: Brief Bioinform. 2024 May 27;25(4):bbae243. doi: 10.1093/bib/bbae243 (PMC11129768; doi:10.1093/bib/bbae243)
Supplement: Supplementary_Table_S1_bbae243 [file supplementary_table_s1_bbae243.docx]

**Supplementary Table S1** The Kendall's coefficient among six scoring methods

| Method | Kendall's coefficient |
| --- | --- |
| ssGSEA vs Viper | 0.437623813 |
| AUCell vs Viper | 0.428032729 |
| AUCell vs JASMINE | 0.422956956 |
| AUCell vs ssGSEA | 0.417629265 |
| UCell vs ssGSEA | 0.40981632 |
| UCell vs Viper | 0.399487836 |
| UCell vs singscore | 0.395079614 |
| singscore vs ssGSEA | 0.392366391 |
| JASMINE vs Viper | 0.391587065 |
| AUCell vs UCell | 0.384435619 |
| UCell vs JASMINE | 0.382581796 |
| singscore vs Viper | 0.371130636 |
| ssGSEA vs JASMINE | 0.370116261 |
| singscore vs JASMINE | 0.357502749 |
| AUCell vs singscore | 0.352813308 |
